# Validation of an immersive virtual reality device accepted by seniors that preserves the adaptive behavior produced in the real world

**DOI:** 10.3389/fbioe.2022.917486

**Published:** 2022-09-02

**Authors:** Lisa Delbes, Nicolas Mascret, Cédric Goulon, Gilles Montagne

**Affiliations:** Aix Marseille Univ, CNRS, ISM, Marseille, France

**Keywords:** fall prevention, elderly, acceptance, immersive virtual reality, head-mounted display, information-movement coupling, real-world vs. virtual reality, adaptive behavior

## Abstract

Falls in the elderly are a major societal issue. Virtual reality appears as a relevant tool to propose gait training programs to prevent the occurrence of falls. The use of a head-mounted display allows overground walking during fully immersive virtual training sessions. Our long-term ambition is to develop gait training programs with a head-mounted display to propose enjoyable and personalized training content for the elderly. Before proposing these programs, several methodological precautions must be taken. The first concerns the supposed similarity of the adaptive behavior produced in the real world and in virtual reality. The second concerns the acceptance of the virtual reality device before and after use. Twenty older adults performed a locomotor pointing task in three conditions including a real-world condition, a virtual-world condition consisting in a replica of the real-world condition, and a virtual condition in which the locomotor pointing task was performed in a different context. From feet positions in relation to the position of a target, gait adaptability behavior was investigated. In line with previous studies, step adjustments (needed and produced) were investigated through a combination of inter-trial and trial-by-trial analyses. The results highlighted that participants adopted the same gait adaptability behavior whatever the type of environment (real vs. virtual). Gait analyses suggested the use of a generic control mechanism based on information-movement coupling. We also demonstrated that older adults accepted the virtual reality device before and after use. With these methodological locks removed, it is now possible to design training programs in virtual reality to prevent falls in the elderly.

## 1 Introduction

In the last decades, Virtual Reality (VR) technologies have been used in fall prevention in the elderly (Plante et al., 2003; Cano Porras et al., 2018; Sakhare et al., 2019), which is a major societal issue. One third of adults over the age of 65 fall at least once each year and half of these have repeated falls (Tinetti et al., 1988; Nevitt, 1989). When a fall occurs, the quality of life for most elderly fallers is lowered because of fear of falling, loss of mobility and independence, social isolation, and institutionalization. Because falls lead to severe injuries, hospitalization, or death among older adults, they are a significant public health issue responsible for a large proportion of health care costs (Ambrose et al., 2013; Burns et al., 2016). Most falls occur during tasks requiring a locomotor displacement in cluttered environments and thus in situations that require the production of adaptive gait (Berg et al., 1997; Robinovitch et al., 2013) and when the displacement adjustments required to deal with environmental constraints are imperfectly performed (Overstall et al., 1977; Nevitt et al., 1991; Ashburn et al., 2008).

Impaired gait adaptability (i.e., the ability to adapt gait in response to obstacles and stepping targets) is associated with a high risk of falls in older adults. Yamada et al. (2011) showed that stepping failures during a multi-target stepping task were associated with an increased risk of falling. Older people at increased risk of falling have reduced gait adaptability (i.e., poor stepping accuracy and more stepping errors) when negotiating unexpected obstacles and stepping targets (Caetano et al., 2016). Moreover, Caetano et al. (2018) compared high- and low-risk groups in a gait adaptability test in which they assessed their ability to adapt gait in response to obstacles and stepping targets under single- and dual-task conditions. They found that impaired gait adaptability is associated with a high risk of falls. All the previous studies suggest that training programs designed to improve gait adaptability are particularly relevant to prevent falls in the elderly (Nørgaard et al., 2021).

During the last decade VR technologies have been shown to be a relevant tool to prevent falls in the elderly (Cano Porras et al., 2018; Khanuja et al., 2018; Keshner et al., 2019). VR technology, defined as a numerical immersive and interactive environment provided by an interface (Burdea, 2003), has been used exponentially in gait training and motor learning in the elderly (Schultheis & Rizzo, 2001; Levin et al., 2015; Howard, 2017; Cano Porras et al., 2018). VR technology qualifies many devices characterized by different levels of immersion. Immersion was defined by Witmer & Singer (1998, *p*. 227) as “a psychological state characterized by perceiving oneself to be enveloped by, included in, and interacting with an environment that provides a continuous stream of stimuli and experiences”. VR technology ranges from low-immersive devices (e.g., standard television monitors and large screens) to high-immersive devices (e.g., head-mounted display (HMD) and Computer-Assisted Virtual Environment (CAVE) systems).

In the field of fall prevention in the elderly, several low-immersive VR devices have been used to propose gait training programs. Low-immersive VR devices that couple a treadmill to a screen displaying specific virtual environments have been widely used to train gait adaptability in the elderly (Mirelman et al., 2020). Improvements in mobility were demonstrated after treadmill training in different realistic virtual environments with this kind of VR device (Shema et al., 2014; Mirelman et al., 2016). More recently, the instrumented treadmill C-mill (Forcelink BV, Culemborg, Netherlands), a treadmill on which virtual 2D targets were projected on the belt, has been used to offer the elderly a wide range of gait adaptability tasks. Van Ooijen et al. (2016) demonstrated that training with the treadmill C-mill led to large improvements in obstacle negotiation performance. Although all these low-immersive VR devices reviewed have so far led to improvements in motor and psychological components (Kamińska et al., 2018; Corregidor-Sánchez et al., 2019; Mirelman et al., 2020) by proposing interactive and personalized exercises increasing the engagement and enjoyment of older adults (Neri et al., 2017; Corregidor-Sánchez et al., 2019; Sakhare et al., 2019), they have several limitations in the field of gait adaptability training. First, the use of a treadmill imposes specific biomechanical constraints on locomotion (e.g., differences in sagittal plane kinematics and other biomechanical outcomes compared with overground locomotion—Van Hooren et al., 2020). In addition, the use of small screens induces lower engagement and enjoyment in training programs in comparison with high-immersive VR devices (Cikajlo and Peterlin Potisk, 2019).

Nowadays, high-immersive VR devices are the most recent VR devices used in gait training. High-immersive VR devices are defined as fully computer-generated environments providing a full field of view using HMD or projection-based systems such as CAVE (Huygelier et al., 2019). These fully immersive VR devices differ from other VR devices by their “high level of ecological validity resulting from the naturalistic sensory-motor interaction between the user and virtual environment” (Tieri et al., 2018, *p*. 9) and “natural interaction with the surrounding environment by using the entire body of the user that becomes, in this way, an active part of the 3D environment” (Tieri et al., 2018, *p*. 2). Delgado and Der Ananian, (2021) recently concluded that it is feasible to use a fully immersive VR device to improve gait and balance among older adults. Weber et al. (2021) also demonstrated that obstacle avoidance training in fully immersive VR could lead to gait adaptability improvements in the elderly. These high-immersive VR devices allow participants to experience virtual environments where they can freely move and walk and perform overground walking. In fall prevention in the elderly, gait adaptability training in fully immersive VR can propose scenarios that mimic real-life tasks (e.g., avoiding an obstacle, stepping over a curb or climbing stairs) with unconstrained locomotion with an enjoyable and personalized training content (Tieri et al., 2018; Sakhare et al., 2019) by adjusting the difficulty level to individual requirements and progression.

To date, no gait training program designed to improve gait adaptability in the elderly (using fully immersive VR without the use of a treadmill) has been conducted despite the many potential advantages of this kind of training program both from a practical and a theoretical point of view. The long-term ambition of our project is precisely to test the effectiveness of this type of program. Now, several methodological precautions must be taken before developing an innovative gait training program. The first concerns the analysis of the supposed similarity of gait adaptability behavior, for a given task, in the real world and in the virtual environment. The second concerns the analysis of the acceptance of the HMD, with which older adults are not familiar. We also analyzed to what extent the context in which a task is performed in VR can have an impact on both gait adaptability behavior and the acceptance of the HMD.

### 1.1 Research questions

First, the overriding concern in relation to our future gait training program is the supposed similarity of gait adaptability behavior between real world and virtual world. In other words, could VR affect the gait adaptive behavior of older adults? This issue is of importance as the beneficial effects of our training program (i.e., good transfer from VR to real life) will be all the more important because the underlying perceptual-motor mechanisms when performing goal-directed locomotion tasks are very similar in the real world and in VR.

Secondly, we must ensure that the participants accept the fully immersive VR technology we plan to use in our future gait training program for the elderly. If the HMD is not accepted by older adults, it is very likely that they will not use it in the end, even if its effectiveness has been validated. That is why our second major challenge concerns the acceptance of technology before use (i.e., *a priori* perception—Hayotte et al., 2020) and its acceptance after use (Alexandre et al., 2018).

#### 1.1.1 Similarity of gait adaptability behavior between real world and virtual world

We must ensure that the gait adaptability behavior of the elderly is the same between the real world and the fully immersive VR environment. Several studies conducted with older adults have shown contradictory results when comparing gait parameters during overground walking in real-world and in virtual environments. Muhla et al. (2020) showed that participants increased their total completion time and their total step number when they performed a Timed Up and Go task, a gold standard in fall risk assessment, in VR. Osoba et al. (2020) found that older adults decreased step length and gait speed and increased step width in VR compared with real-world, while Janeh et al. (2018) found no differences on these two gait parameters. Nevertheless, these two last studies found similar results concerning step width, with an increased width in VR compared with the real world.

It is worth noting that most of the studies which compare gait behavior produced in real-world and virtual environment have focused on very simple locomotion tasks in which participants are not asked to interact with their environment. This makes the study of adaptive behavior difficult, if not impossible. Even if gait parameters (e.g., step length and gait speed) are different between real world and fully immersive VR, gait adaptability behavior (i.e., the capacity to modulate gait parameters to deal with environmental constraints) is not necessarily affected. In our future gait training program in VR, older adults will perform several goal-directed locomotion tasks (e.g., obstacle interception or avoidance, stepping targets) previously used to study gait adaptability behavior in the elderly (Weerdesteyn et al., 2008; Caetano et al., 2016; Van Ooijen et al., 2016). In response to environmental challenges, appropriate gait adjustments are required. Regarding comparison of gait adaptability behavior between real and fully immersive virtual environments, several studies have been conducted with young adults during gait-specific tasks (Fink et al., 2007; Agethen et al., 2018; Buhler and Lamontagne, 2018). In these studies, young adults performed obstacle avoidance during overground walking in real world and VR, where the virtual environment was a replica of the real one. Young adults adopted a more conservative strategy in virtual environment (VE) (i.e., slower gait speed and larger clearance).

However, the study of gait adaptability behavior has often been limited to the analysis of gait parameters reflecting only the participant behavior (i.e., without indicators of gait adjustments related to the environment). In studies showing how gait adaptability training improves obstacle avoidance skills and prevents falls, the study of gait adaptability behavior was reduced once again to these same gait parameters. Mirelman et al. (2016) based their gait adaptability behavior analyses on gait speed and variabilities, and foot clearance during obstacle negotiation training. During obstacle avoidance training, Van Ooijen et al. (2016) investigated gait adaptability through the assessment of gait speed and avoidance performance (i.e., success rate), while Weerdesteyn et al. (2008) studied the avoidance reaction time, the distribution of avoidance, and spatial avoidance parameters such as distance and foot clearance. Moreover, Timmermans et al. (2019) conducted gait adaptability behavior analysis only on task performance during their seven walking-adaptability tasks. Additional levels of analysis are required to study gait adaptability behavior. In the studies reviewed in the previous section, the study of gait adaptability behavior was based on gait parameters (e.g., gait speed or clearance performance) but gait adjustments allowing participants to deal with environmental challenges were imperfectly investigated. A precise characterization of the evolution of the state of the agent-environment system when performing goal-directed locomotion tasks would lead to a better investigation of underlying mechanisms. According to the postulates of ecological psychology (Gibson, 1979; Warren, 1998) gait adjustments are based on a continuous coupling between information and movement. This tight coupling between information and movement has been very well described in tasks in which the positions of the feet and/or the body must be controlled in relation to either stationary or moving objects in the surroundings (e.g., Lee et al., 1982; De Rugy et al., 2000; Montagne et al., 2000; Van Andel et al., 2018a). In an everyday-life context, the mechanisms underlying gait adjustments have been analyzed in two studies in which subjects had to step onto a curb (Van Andel et al., 2018a) and step over an obstacle (Cornus et al., 2009). In these two studies, participants’ gait adjustments were analyzed by a method proposed by Montagne et al. (2000) based on the recording of successive feet positions in relation to the target to be pointed at. Based on the combination of inter-trial and trial-by-trial analyses, this method makes it possible to determine step adjustments (needed and produced). Taken together, the results obtained in these studies (e.g., Montagne et al., 2000; De Rugy et al., 2001, 2002; Cornus et al., 2009; Van Andel et al., 2018a) suggest the use of a control mechanism 1) based on a tight coupling between information and movement and 2) leading to successive adjustments depending on the state of the agent-environment system.

To sum up, the first issue addressed in this study concerns the similarity of gait adaptability behavior between real-world and virtual environments. We had to ensure that gait adaptability behavior in these two types of environments is similar before proposing our training program to elderly people. The gait adaptability behavior of older adults was investigated by a method allowing precise analysis of the successive step adjustments made to deal with task constraints in a locomotor pointing task.

#### 1.1.2 Acceptance before and after use of the VR technology

The Technology Acceptance Model (TAM) (Davis, 1989; Venkatesh & Davis, 2000; Venkatesh & Bala, 2008) is the most widely used model to study acceptance of a technology. The TAM postulates that intention to use a technology is positively predicted by its perceived usefulness and its perceived ease of use. In the field of VR, perceived enjoyment (i.e., the perceived degree of enjoyability when using the technology) was also integrated into the TAM as a factor predicting the intention to use VR technology (Manis & Choi, 2019).

In the field of fall prevention in the elderly, the TAM has been recently used to study acceptance before use of an HMD device intended to prevent falls among the elderly (Mascret et al., 2020). The study highlighted that the intention to use an HMD was positively predicted by perceived usefulness, perceived enjoyment, and perceived ease of use and that perceived usefulness of this HMD was negatively predicted by fall-related self-efficacy (i.e., the perceived level of confidence of an individual when performing daily activities without falling) and positively predicted by self-avoidance goals (i.e., having a physical activity to avoid physical regression). However, this study was conducted using self-reported questionnaires following a presentation of the device based on a written text and several pictures. Consequently, the dynamic of its acceptance (after use) throughout gait training programs has not yet been studied. That is why the present study investigated acceptance of the VR-HMD before using it and the dynamic of its acceptance after using it during a specific gait-specific task in fully immersive VR. Among older adults, Huygelier et al. (2019) demonstrated that the participants’ acceptance of an HMD increased after a first use of a few minutes, but it was not studied in the context of fall prevention with a gait adaptability task. In the present study, the aim is twofold concerning acceptance: to investigate its first level before use and whether its level changes after use.

Moreover, in older adults, some extended TAMs have been used to investigate the acceptance of VR devices including user experience (Syed-Abdul et al., 2019) which is associated with the quality of the immersive user experience in VR (Mütterlein & Hess, 2017). User experience may be influenced by cybersickness and sense of presence. Cybersickness quantifies motion sickness induced by discrepancies between visual, vestibular, and proprioceptive information (Robillard et al., 2003). Sense of presence quantifies subjective experience of being in a virtual environment (Witmer & Singer, 1998). Sagnier et al. (2020) demonstrated that cybersickness negatively predicts intention to use an HMD, while presence is not a significant predictor. But their study was conducted with young adults during an aeronautical assembly task and did not concern the field of gait training among the elderly. Given that these two feelings can affect the user experience (Tieri et al., 2018; Weech et al., 2019), we must investigate cybersickness and sense of presence in fully immersive VR in this present study.

To sum up, the second issue addressed in this study was to ensure that the participants accept the VR-HMD before use and to know whether the acceptance changes after use. We also investigated their subjective experience through the study of cybersickness and sense of presence.

#### 1.1.3 Sub-challenge

In this study, we also wanted to investigate the influence of the virtual context on gait behavior. VR offers the possibility of developing a wide range of virtual environments. Diversified virtual environments can offer more enjoyable and engaging exercises in our future gait training program in fully immersive VR. The visual context in VR should be considered. Contextual realism can modify the ecological validity of a simulated environment (Kuliga et al., 2015; Joseph et al., 2020). Birenboim et al. (2021) recommended pretesting VE design to ensure high ecological validity in terms of behavior and wellbeing in fully immersive VR. Moreover, highly realistic VR environments led to greater positive affective and serenity responses compared with low-realism environments (Newman et al., 2022).

It is known that the visual context can affect human behavior. Studies have shown that an outdoor environment leads to greater relaxation (Anderson et al., 2017), restoration (Rogerson et al., 2016) and level of vigor (Yu et al., 2018) and to a faster and more complete recovery from stress (Yin et al., 2020) than an indoor environment. Moreover, Lourenço et al. (2008) showed that motivation was greater in the more enjoyable VR environment (i.e., more interesting visual and auditory interface) during a reaching task. The manipulation of the visual context may also impact gait behavior in the elderly. That is why, before proposing our gait training in different virtual environments, we must ensure that diversified virtual environments preserve gait adaptability behavior. In this present study, two different virtual environments were computed: a corresponding one (i.e., a replica of the real-world set-up) and a non-corresponding one (i.e., outdoor environment). Our non-corresponding virtual environment had the same dimensions and the same informational content as our corresponding virtual environment. In our gait-specific tasks, the available information in the visual field should be the same so as to strictly compare gait adaptability behavior in the two conditions.

### 1.2 Hypotheses

In summary, before developing our fully immersive VR training program to prevent falls in the elderly, several methodological precautions must be taken. First, we must ensure that gait adaptability behavior in the elderly is the same whatever the environment (real world vs*.* fully immersive VR) during a locomotor pointing task. The use of an immersive VR device that preserves natural gait leads us to hypothesize that gait adaptability behavior of older adults should not be affected by the type of environment (H1). We also need to check the acceptance of the HMD (before and after its use) in the elderly during a locomotor pointing task in a fully immersive VR environment. We hypothesize that the HMD should be accepted by older adults before its use (H2) and even more after its use (H3). We also hypothesize a low level of cybersickness (H4) and a high level of sense of presence (H5) in both virtual conditions. Finally, we hypothesize that manipulating the context in which the task is performed while leaving the informational content of the environment unchanged should not affect behavior when comparing gait adaptability behavior produced in the two virtual conditions (H6).

## 2 Methods & materials

### 2.1 Experiment

#### 2.1.1 Participants

Twenty older adults (mean age = 68.6 years, SD = 3.4 years) volunteered to take part in the experiment. Participants had normal or corrected-to-normal vision and were included in the study if they were able to walk independently, without a cane, crutch, or walker, and without help from another person. These participants had no neurological, vestibular, cardiac, pulmonary, or orthopedic disorders. These potential disorders were self-reported by the participants before the beginning of the experiment. Participants had a Montreal Cognitive Assessment (MoCA) score between 26 and 30. The MoCA cut-off score was selected based on selective criteria for intact cognition (Nasreddine et al., 2005). Participants should have never used an HMD before. Participants were not informed about the precise purpose of the study. All participants gave informed consent. The study was conducted in line with the ethical guidelines of the national ethics committee (Ethical Committee for Research no. IRB00012476-2020-25-03-51), as well as in compliance with the Declaration of Helsinki for human research and the international principles governing research on humans.

#### 2.1.2 Instruments and material

**FIGURE 1:**
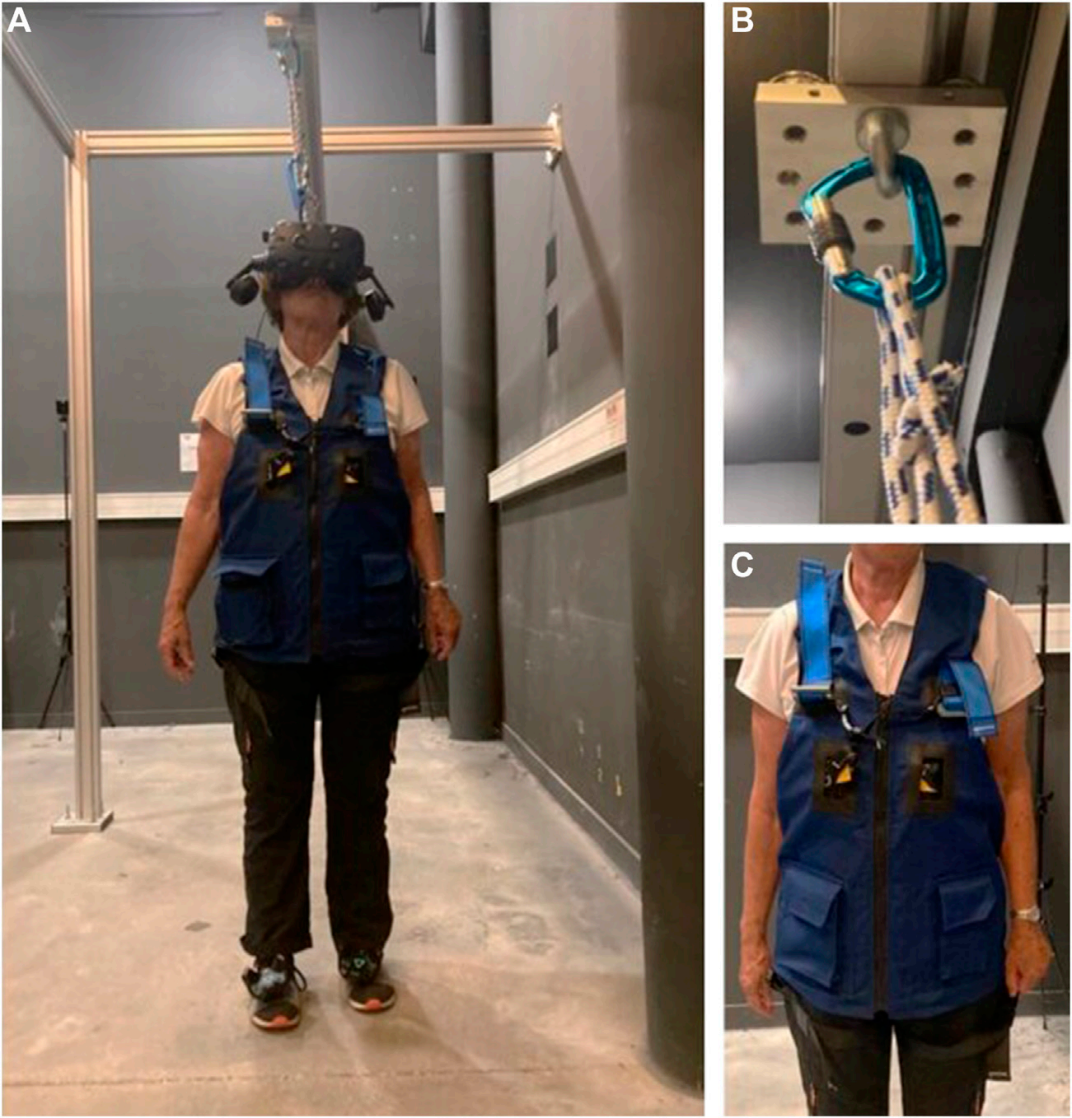
Illustrations of the test equipment. **(A)** Older adult performing the task. A wireless HTC Vive Pro^®^ was used to display the VR environment. Two HTC Vive Pro^®^ 2.0 trackers (placed on the feet) were used to record the positions of the feet and display virtual 3D shoes in the VR. The security system is composed of **(C)** a safety harness connected to **(B)** an in-ceiling rail by a lanyard.

In each condition, the participant was equipped with a security system consisting of a safety harness connected to an in-ceiling rail by a lanyard (Figures 1B,C). The in-ceiling rail only allowed rectilinear movements within a steel structure. In all the conditions, participant’s feet positions were recorded by two HTC Vive Pro^®^ foot trackers during each locomotor pointing task (Figure 1A).

##### 2.1.2.1 VR system: HTC vive pro^®^

For the virtual conditions, the VR system consists of a wireless VR-HMD which displayed a VE, four trackers, and four lighthouse lasers. A computer with 2.8 GHz Intel^®^ Core i7 processor, 8 GB of main memory, and NVIDIA^®^ GeForce GTX 1070 graphics card was used to run the software ICE^®^. ICE^®^ (Imagine, Create and Experiment) is a free 3D software developed in C++ and OpenGL for researchers who are not familiar with programming (trello link about ICE: https://trello.com/b/EtNCNrZH/ice). It is composed of “ICE_Designer” tool used to build 3D scenes with the implementation of tracking systems and of “ICE_Protocol” tool used to display the scene with the experimental settings (which scenes, in what order, participants’ settings, initial conditions … ) and to save experimental data. A wireless HTC Vive Pro^®^ HMD was used to display a VE for the participant. This headset has a resolution of 1440 × 1600 pixels per eye (refresh rate of 90 Hz) and a diagonal field of view of 110°.

**FIGURE 2:**
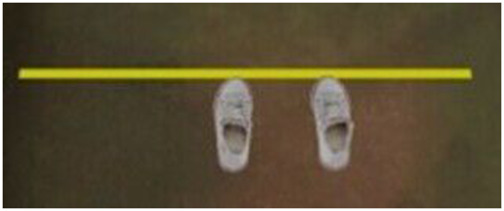
Virtual 3D shoes representing participants’ feet in the virtual environment. The size of the virtual 3D shoes was adjusted to the size of the participant’s feet.

Participants’ feet were presented in the VE by two virtual 3D shoes (Figure 2) with the two HTC Vive Pro^®^ trackers placed on the feet (the same ones which recorded feet positions). For each participant, the size of the virtual 3D shoes was adjusted to the size of the participant’s his/her feet in the settings of “ICE_Protocol”. A supplementary tracker, placed on the floor, was used to display a blue target (120 cm wide and 2.5 cm long) and a yellow line (120 cm wide and 2.5 cm long), which marked the start area.

Four lighthouse lasers, with a span of 120° in each direction, were used to detect the position and orientation of the HMD and of the four trackers at an update rate of 120 Hz with sub-millimeter precision and accuracy for position data^1^.

#### 2.1.3 Protocol

The first purpose of this study was to record participants’ gait parameters (by measuring feet positions) while performing a locomotor pointing task in three experimental conditions: one real-world condition (RWC) and two virtual reality conditions (VRC).

##### 2.1.3.1 Task

**FIGURE 3:**
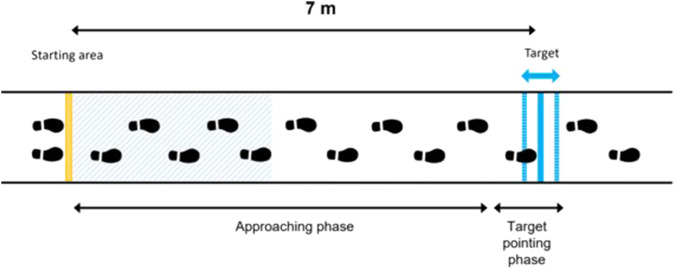
Diagram of the locomotor pointing task consisting of an approaching phase and a target-pointing phase. Participants were instructed to place themselves in the starting area and to start walking with the right leg. For each trial, participants were asked to place their right foot tip as close as possible to the lower extremity of the target while walking at a normal pace and continuing to walk subsequently. The position of the target was manipulated unbeknownst to the participants (3 different target positions: 0 ± 20 cm). The blue hatched area represents the area for calculating the average gait speed (i.e., the first five steps).

The task performed in this study was a locomotor pointing task (Figure 3) which is paradigmatic when studying the mechanisms underlying goal-directed locomotion (Lee et al., 1982). The locomotor pointing task consists of two phases: an approaching phase and a target-pointing phase as in the studies of Montagne et al. (2000), De Rugy et al. (2000), Cornus et al. (2009), and Van Andel et al. (2018a). Participants were instructed to place themselves in the starting area and to start walking with the right leg. For each trial, participants were asked to place their right foot tip as close as possible to the lower extremity of the target while walking at a normal pace and continuing to walk subsequently. After each trial, participants had to return to the starting position walking at a normal pace. To avoid locomotor regularities and prevent any calibration of the distance being covered, the position of the target location was manipulated unbeknownst to the participants (3 different target positions: 0 ± 20 cm) and presented in a randomized order (6 or 7 trials for each target position condition). Instructions concerning the experimental task were given in a short explanatory video. Afterwards, participants could ask the experimenter questions and instructions could be reformulated if necessary.

##### 2.1.3.2 Experimental conditions

**FIGURE 4:**
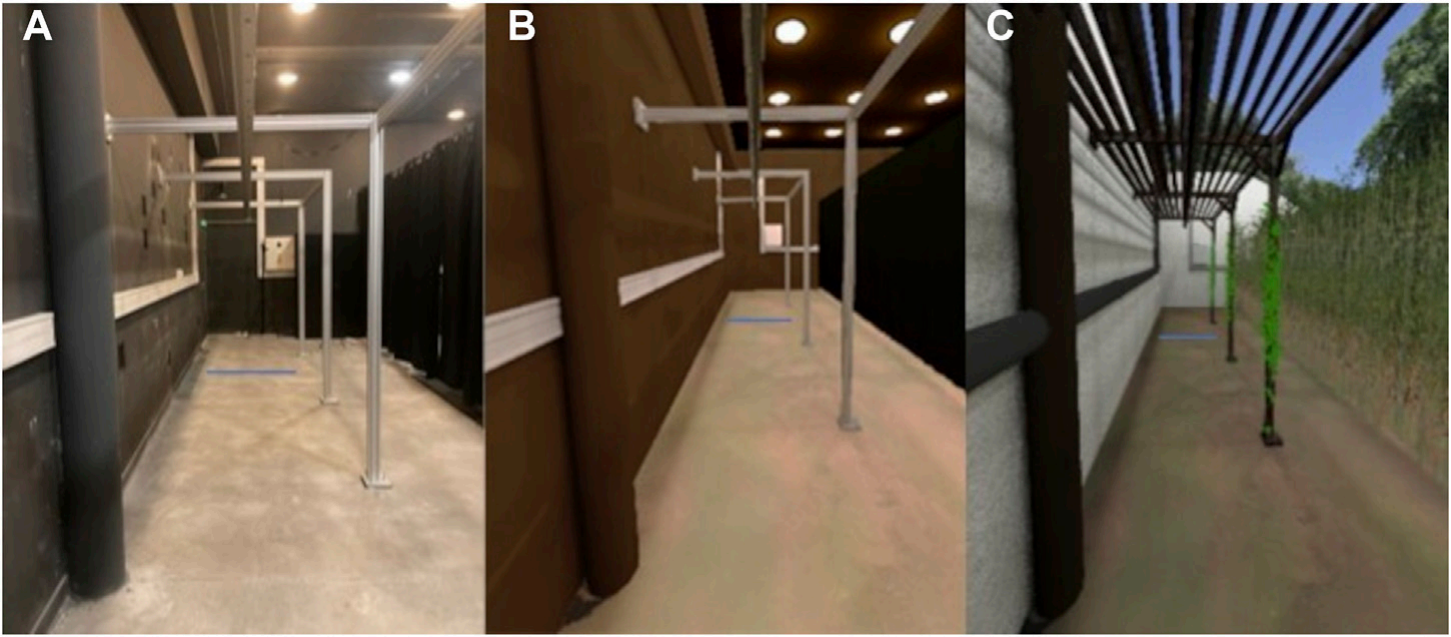
Experimental conditions. **(A)** Picture of the “real-world condition”. **(B)** Screen shot of the “corresponding virtual reality condition”. **(C)** Screen shot of the “outdoor virtual reality condition”.

For the RWC, participants had to walk in the experimental room (Figure 4A). For the VRC, two VEs were created. For the “corresponding VR” condition, the VE consisted of a reconstitution of the experimental room (Figure 4B). For the “outdoor VR” condition, the VE consisted of an outdoor environment (Figure 4C) giving rise, for a given displacement, to an identical optical flow to the other two conditions. In the VRC, participants walked in the VE wearing the VR-HMD HTC Vive Pro^®^.

##### 2.1.3.3 Procedure

At the beginning of the experiment, the participants watched a short video with pictures of the VR device used (HMD), to present the VR set-up and its scientific applications. Afterward, they were equipped with the security system and HTC Vive Pro^®^ trackers.

The experimental conditions were presented in a randomized order. Between each experimental condition, participants had a forced rest period of 10 minutes to minimize fatigue accumulation in experimental conditions.

**FIGURE 5:**
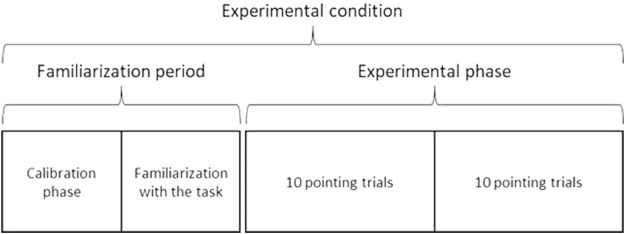
For the three experimental conditions, the experiment was composed of a familiarization period followed by an experimental phase. The familiarization period was split into a calibration phase and a familiarization with the task. The calibration phase consisted of 2 minutes of free walking in the environment. It was designed to allow participants to become familiar with their displacement in the VR environment. The familiarization with the task consisted of successive pointing trials. It was designed to make sure that each participant was familiar with the pointing locomotion task requirements and that they understood the task instructions. After the familiarization period, the experimental phase consisted of two blocks of 10 pointing trials per condition.

For the three experimental conditions, the experiment was composed of a familiarization period followed by an experimental phase (Figure 5). The familiarization period was split into a calibration phase and a familiarization with the task. The calibration phase consisted of 2 minutes of free walking in the environment (without performing the pointing task). It was designed to allow participants to become familiar with their displacement in the environment. More precisely, the calibration phase was designed to allow each participant to get used to the VR interface and to ensure that participants become familiar with virtual 3D shoes representing their feet during the locomotion in VR. Afterward, the familiarization with the task consisted of successive locomotor pointing trials (minimum five). A success rate was calculated from the ratio of pointing accuracy lower than 10 cm over the last five trials. The familiarization with the task stopped when the success rate of the participants was greater than 80%. It was designed to make sure that each participant was familiar with the pointing locomotion task requirements and that they understood the task instructions. After the familiarization period, the experimental phase consisted of two blocks of 10 pointing trials per condition. Twenty trials by condition allow a substantial repetition of the task to have a good representation of the gait adaptability behavior, but it should not be large in order to avoid fatigue over the different conditions.

### 2.2 Outcome variables

#### 2.2.1 Gait parameters

**FIGURE 6:**
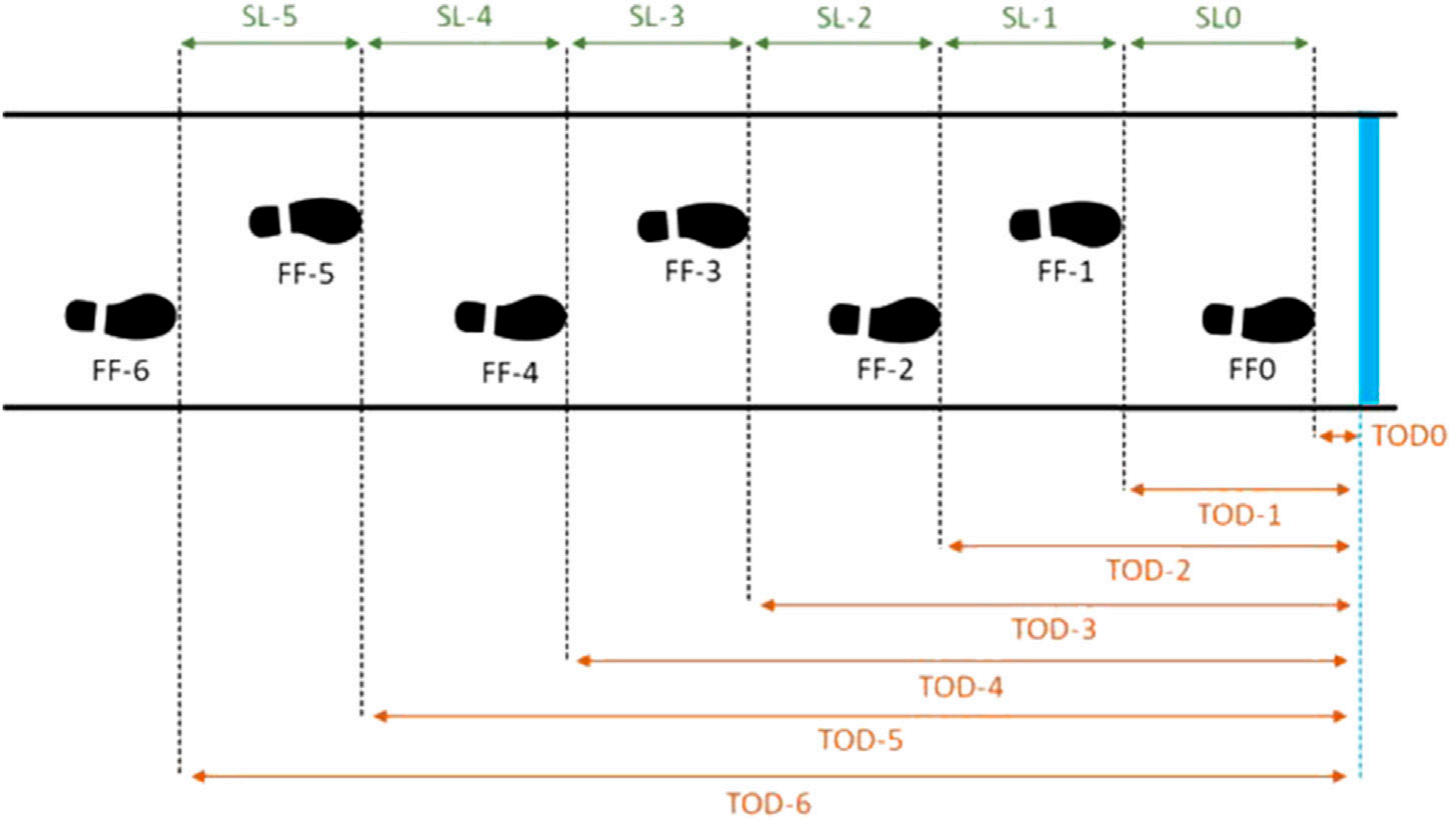
Schema of gait parameters calculation during the locomotor pointing task. The blue line represents the target. Footfall i (FFi) coding: the footfall of pointing is coded FF0. Step length (SLi) coding for each footfall i. Toe-obstacle distance (TODi) coding for each footfall i.

Gait speed, step lengths, and toe-obstacle distances were computed from the feet positions recorded by the feet trackers (Figure 6).

From these computations, several gait parameters were computed following the methodology used in previous studies (De Rugy et al., 2000; Montagne et al., 2000; Cornus et al., 2009; Van Andel et al., 2018a). Gait parameters were computed using MATLAB^®^ (version R2017b, The MathWorks, Inc., USA). A step corresponded to two consecutive foot contacts (i.e., footfalls) of two different legs.

Several types of gait analysis were performed. A pointing accuracy analysis and a gait speed analysis were first performed. Following the procedure used by Montagne et al. (2000), Cornus et al. (2009), and Van Andel et al. (2018a), inter-trial and trial-by-trial analyses were also performed. In the inter-trial analysis, the inter-trial standard deviations of both the toe-obstacle distances and the step lengths over the target approach were computed. Two types of analysis were performed in the trial-by-trial analysis: an inter-step number analysis and an intra-step number analysis.

##### 2.2.1.1 Gait speed analysis

Separately for each participant, the average gait speed for the first five steps of each trial was computed. The standard deviation (within-subject inter-trial) of the gait speed was also calculated.

##### 2.2.1.2 Pointing accuracy analysis

The pointing footfall was defined as footfall 0 (FF0). The toe-obstacle distance of FF0 represented the pointing accuracy, that is the distance between the toe and the target at last footfall. Negative values were related to pointing performances before the target (i.e., undershoot) and positive values were related to pointing performances after the target (i.e., overshoot).

The absolute and constant pointing errors were computed to measure the pointing performance (Schmidt and Lee, 1982). The following formulae explain how they are computed, with *n* the number of participants, *xi* the position of pointing *i* and *T* the position of the target.

The absolute pointing error (APE) reflects the overall accuracy without considering the direction of the error (see Eq. 1). The APE is the mean absolute value of the difference between the toe of the foot and the target at the last footfall.

$$APE=\frac{\left(\sum \left|xi-T\right|\right)}{n}$$ (1)

The constant pointing error (CPE) reflects the direction of the pointing errors (see Eq. 2). The CPE is the signed mean difference between the toe of the foot and the target at the last footfall (negative when the toe was before the target and positive when the toe was after the target).

$$CPE=\;\frac{\left(\sum xi-\;T\right)}{n}$$ (2)

##### 2.2.1.3 Inter-trial analyses

In the inter-trial analyses, the standard deviation of the toe-obstacle distances and the standard deviation of the step lengths were measured. Separately for each participant, the toe-obstacle distance and the step length were computed for each footfall. For each footfall, the standard deviation (within-subject inter-trial) of the toe-obstacle distance and of the step length were calculated across all trials. Following previous studies in locomotor pointing, we can anticipate that during the approach to the target the step length variability increases while the toe-obstacle distance variability decreases (e.g., Lee et al., 1982; Montagne et al., 2000). The modulation of step lengths in the final steps (i.e., increase in the standard deviation of step lengths) to perform an optimal foot positioning tends to be increasingly accurate when approaching the target (i.e., decrease in the standard deviation of toe-obstacle distances). This pattern of compensatory variability underlines the need to correct the current foot position by changes in step length to perform an accurate pointing (Cornus et al., 2009; Van Andel et al., 2018a).

##### 2.2.1.4 Trial-by-trial analyses

In the trial-by-trial analyses, each trial was analyzed separately. An inter-step number analysis and an intra-step number analysis were performed.

For the inter-step number analysis, an average pattern of step length was calculated from the mean step lengths of all 30 trials for each participant. Following Montagne et al. (2000), a threshold of 4% is established on both sides of the average pattern of step length. For each participant and each trial, adjustments were identified by comparing each step length with the average pattern of step length. Thereby, a step was characterized as a lengthening step if its length was greater than the average pattern of step length and if the difference exceeded the threshold. A step was characterized as a shortening step if its length was smaller than the average pattern of step length and if the difference exceeded the threshold.

A trial was described as regulated if at least one of its steps was characterized as a lengthening or shortening step. Three categories of regulated trials were defined: 1) lengthening trials where only lengthening steps were performed, 2) shortening trials where only shortening steps were performed, and 3) mixed trials with a combination of lengthening and shortening steps.

The intra-step number analysis was conducted to compute the amount of adjustments needed (in relation to toe-obstacle distances) and the amount of adjustments produced (in relation to step lengths) for each footfall. This analysis allowed us to determine the relationship between these two parameters and thereby to assess the strength of information-movement coupling.

The amount of adjustments needed (AN) represents the difference between the current toe-obstacle distance and the average toe-obstacle distance for that footfall. The amount of adjustments needed for footfall i (AN*i*) was the difference between the toe-obstacle distance for footfall i (TTD*i*) and the average of the toe-obstacle distance of footfall i (ATTD*i*) (Eq. 3).

ANi = TTDi−ATTDi (3)

The amount of adjustments produced (AP) represents the difference between the current step length and the average of the step length for that footfall. The amount of adjustments produced at footfall i (AP*i*) was the difference between the step length of footfall i (SL*i*) and the average step length for footfall i (ASL*i*) (Eq. 4).

APi = SLi−ASLi (4)

The amount of adjustments needed for the current footfall is assumed to be related to the amount of adjustments produced at the next footfall. The current difference of toe-obstacle distance (compared with the average of the toe-obstacle distance) at the current footfall should be corrected by modulation of step length at the following footfall. This relationship is used to assess the strength of information-movement coupling (De Rugy et al., 2000; Montagne et al., 2000; Cornus et al., 2009).

#### 2.2.2 Psychological variables

##### 2.2.2.1 Acceptance of the VR-HMD

Acceptance was measured at the beginning of the experiment (after the short video with pictures of the HMD) using self-reported questionnaires to quantify participants’ acceptance of the VR-HMD before use (Appendix A), in line with the procedure of Huygelier et al. (2019). After the second VRC, they once again completed the acceptance questionnaire to quantify their acceptance of the VR-HMD after use (Appendix B). Acceptance of the VR-HMD HTC Vive Pro^®^ before and after use was assessed through four variables: perceived usefulness, perceived ease of use, perceived enjoyment, and behavioral intention to use (Davis, 1989; Davis & Venkatesh, 1996; Manis & Choi, 2019). Participants answered the three items per variable on a Likert scale from 1 (*strongly disagree*) to 10 (*strongly agree*): perceived usefulness (e.g., “*I believe using VR hardware would help me be more effective in my locomotion*”), perceived ease of use (e.g., “*I believe using VR hardware would be easy for me*”), perceived enjoyment (e.g., “*I believe I would find using VR hardware enjoyable*”), and behavioral intention to use (e.g., “*I intend to use VR hardware within the foreseeable future*”).

Internal consistency was good for each variable, with McDonald’s omegas ranging from 0.82 to 0.99. McDonald’s omegas were used because Cronbach’s alphas have a tendency to over-estimate or under-estimate reliability (Dunn et al., 2014). McDonald’s omegas for each variable, groups, and measurement times are provided in Table 1.

**TABLE 1:** McDonalds’s omega, mean and standard deviation (SD) of the four variables of acceptance at the beginning of the experiment (T1) and after the second virtual reality condition (T2): perceived usefulness (PU), perceived ease of use (PEOU), perceived enjoyment (PE), and behavioral intention to use (BIU).

	T1		T2	
	McDonald’s omega	Mean (SD)	McDonald’s omega	Mean (SD)
**PU**	0.97	7.42 (2.53)	0.97	6.37 (2.97)
**PEOU**	0.95	8.02 (2.12)	0.94	9.12 (1.00)
**PE**	0.99	8.22 (2.55)	0.82	8.97 (1.61)
**BIU**	0.98	6.52 (3.01)	0.99	7.05 (3.30)

##### 2.2.2.2 Cybersickness and sense of presence

Cybersickness and sense of presence were measured after the familiarization period of each virtual condition and at the end of the two pointing blocks of each virtual condition by oral questionnaires (Robillard et al., 2003). To quantify cybersickness, the experimenter asked the participants to rate verbally on a 10-point Likert scale (“*How unwell is the virtual reality making you feel?*”) from 1 (“*do not feel unwell at all*”) to 10 (“*feel very unwell*”). To quantify the sense of presence, the experimenter asked the participants to rate verbally on a 10-point Likert scale (“*How much do you feel that you really are present in the virtual environment you see?*”) from 1 (“*not at all present*”) to 10 (“*completely present*”). In this study, a short verbal 10-point Likert scale was used for repeated measurements of cybersickness and sense of presence because it is an optimal scale of response with older adults, and it has higher reliability, validity, and discrimination (Wee et al., 2008). A short scale is indeed relevant for repeated measurements as in our study.

**FIGURE 7:**
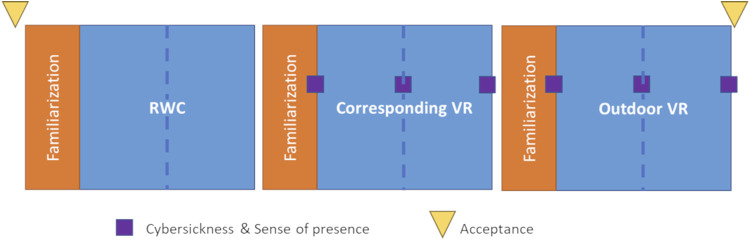
Experimental procedure in a specific order: real-world condition (RWC), corresponding virtual reality (VR) and outdoor virtual reality (VR). The experimental conditions were presented in a randomized order. The three conditions consisted of a familiarization period (in orange) and an experimental phase (in blue). The dotted blue line represents the two blocks of 10 pointing trials per condition. During the experimental phase of each condition, feet positions were recorded for each trial to compute gait parameters. Cybersickness and sense of presence were measured at the end of the familiarization period and at the end of the two pointing blocks of each virtual condition. Acceptance was measured at the beginning of the experiment to quantify acceptance before use, and once again, after the second RVC, to quantify their acceptance after use.

The experimental procedure with the outcome psychological variables is summarized in Figure 7.

### 2.3 Statistical analyses

The statistical software JASP (Version 0.14.1, 2021) was used to run statistical analyses. The statistical level of significance was set at *α* = 0.05. Non-parametric tests were used when the distribution of the data was non-normal.

For gait parameters, between conditions, differences in strategy for the participants were evaluated using non-parametric Friedman tests for gait speed, pointing accuracy, patterns of standard deviation of toe-obstacle distances and of step lengths, proportion of regulated trials, and adjustment strategies. A linear regression was calculated to assess the relationship between the amount of adjustments needed at a certain footfall and the amount of adjustments produced at the following footfall. Friedman tests were used for footfalls two by two for each condition to assess the onset of step adjustments (based on standard deviations).

For psychological variables, one-sample *t* tests were conducted on each variable to see if they differed significantly from the mean of the scale (i.e., 5.5). Then, for acceptance, between-times differences were evaluated using non-parametric Wilcoxon signed-rank tests.

## 3 Results

### 3.1 Gait parameters

Several analyses were conducted to study between-conditions differences in the participants’ gait adaptability behavior.

#### 3.1.1 Gait speed analysis

For the gait speed, the Friedman test revealed no significant differences between conditions (*χ*2 (6) > 20.569, *p* = 0.153). The participants walked at 1.07 m/s (SD = 0.02 m/s) in RWC, at 0.96 m/s (SD = 0.02 m/s) in corresponding VR, and at 0.98 m/s (SD = 0.02 m/s) in outdoor VR. They thus walked almost at the same speed whatever the condition.

#### 3.1.2 Pointing accuracy analysis

**FIGURE 8:**
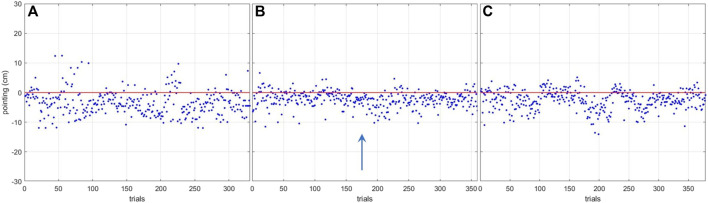
Global pointing performances in the three conditions. **(A)** real-world condition. **(B)** corresponding virtual reality condition. **(C)** outdoor virtual reality condition. The red line marks the lower extremity of the target. The blue arrow indicates the direction of the locomotor displacement.

Pointing accuracy analysis was based on global pointing performance across all the trials (Figure 8).

To quantify the accuracy, the APE and the CPE of the participants were computed across the different conditions.

For the APE, the Friedman test revealed a significant difference between conditions (χ2 (2) > 20.632, *p* <0 .001). Conover’s Post Hoc test revealed a difference between the RWC condition and the VRC (*p* <0 .001 and *p* = 0.001 for corresponding VR and outdoor VR, respectively) but no difference between the VRC (*p* = 0.521). The participants’ APEs were 4.53 cm (SD = 1.43 cm) in RW, 2.78 cm (SD = 0.86 cm) in corresponding VR, and 3.20 cm (SD = 1.66 cm) in outdoor VR. The participants had a lower absolute pointing error in VRC than in RWC.

For the CPE, the Friedman test revealed a significant difference between conditions (χ2 (2) > 8.316, *p* <0 .016). Conover’s Post Hoc revealed a difference between the RWC condition and the VRC (*p* = 0.009 and *p* = 0.042 for corresponding VR and outdoor VR, respectively) but no difference between the VRC (*p* = 0.521). Participants’ CPEs were −3.52 cm (SD = 1.84 cm) in RWC, −2.47 cm (SD = 0.98 cm) in corresponding VR, and -2.51 cm (SD = 2.11 cm) in outdoor VR. The results showed that participants tend to place their feet before the target in the three conditions but that the performance was better in VRC than in RWC.

#### 3.1.3 Inter-trial analyses

**FIGURE 9:**
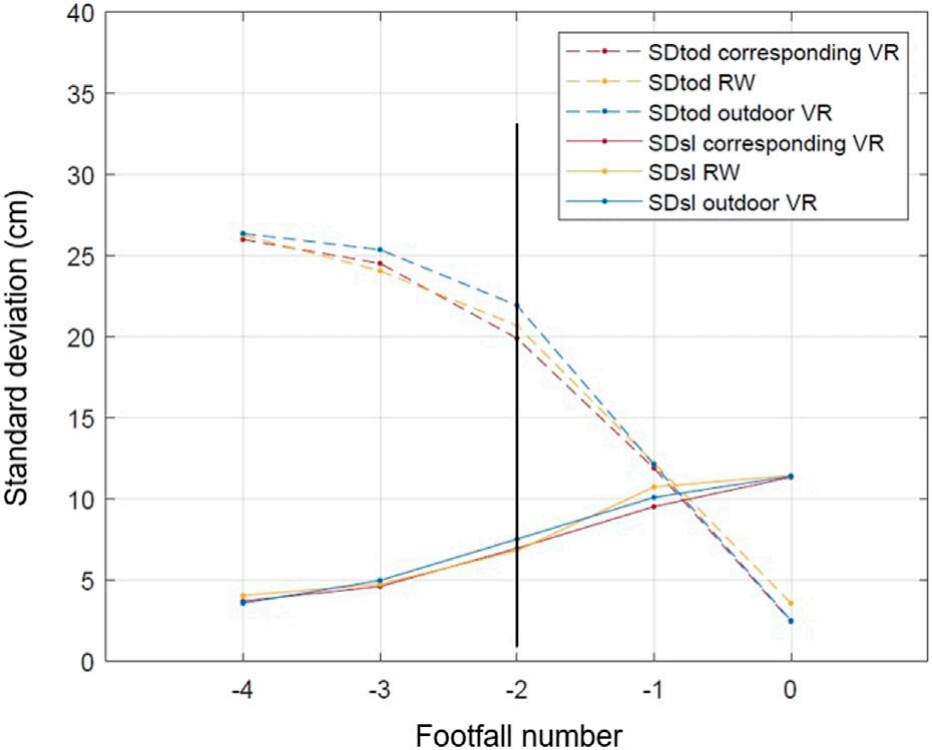
Patterns of the standard deviation of toe-obstacle distances and of step lengths as functions of footfall number. The black vertical line marks the onset of step adjustments at footfall −2. Footfall 0 is the pointing footfall. SDtod: standard deviation of toe-obstacle distances; SDsl: standard deviation of step lengths; VR: virtual reality; RW: real world.

Standard deviation of toe-obstacle distances (SD_tod_) and the standard deviation of step lengths (SD_sl_) were computed as a function of footfall number in the three experimental conditions (Figure 9). No difference was found between the patterns of the SD_tod_ and the SD_sl_ in the three conditions (all *p*s > 0.05). In the three conditions, at the beginning of the target approach, the SD_tod_ was high (26.11 cm on average) and decreased through the approaches with an average value of 3.64 cm at the last footfall (i.e., pointing footfall). Conversely, in the three conditions, the SD_sl_ was low (4.12 cm on average) at the beginning of the target approach and increased through the approaches with an average value of 11.51 cm at the last footfall (i.e., the pointing footfall).

Following previous studies (Berg et al., 1994; Montagne et al., 2000; Cornus et al., 2009) the onset of step adjustments (i.e., the footfall number at which gait regulation was initiated) was established as the footfall at which the SD_tod_ started to decrease and the SD_sl_ started to increase. The results showed that the onset of step adjustments was at footfall −2 on average whatever the condition. The pattern of the SD_tod_ decreased significantly at footfall −2 compared with footfall −3 (all *p*s <0.05) and SD_sl_ increased significantly at footfall −2 compared with footfall −3 (all *ps* <0 .05). The results showed that the participants initiated their step adjustments two steps before the target on the three conditions to perform an optimal foot positioning.

#### 3.1.4 Trial-by-trial analyses

##### 3.1.4.1 Inter-step number analysis

For the proportion of regulated trials, the Friedman test revealed no significant differences between conditions (*χ*2 (2)>0.758, *p* = 0.685). The proportion of regulated trials was 88.31% (SD = 7.45%) in RWC, 87.89% (SD = 7.87%) in corresponding VR and 88.93% (SD = 6.98%) in outdoor VR. Among regulated trials, the distribution of adjustment strategies of step length (mixed, lengthening, and shortening) was computed across the three conditions (Table 2). The Friedman test revealed no significant differences between conditions (*χ*2 (2) > 0.111, *p* = 0.946; *χ*2 (2) > 4.388, *p* = 0.111; *χ*2 (2) > 4.388, *p* = 0.111; for mixed, lengthening, and shortening trials respectively). In the three conditions, the participants adopted mostly mixed trials (almost half of trials).

**TABLE 2:** Distribution of adjustment strategies of step length (mixed (MT), lengthening (LT), and shortening (ST) trials) in the three experimental conditions (RWC: real-world condition; VR: virtual reality).

	RWC	Corresponding VR	Outdoor VR
**MT (%)**	45.47	49.10	47.02
**LT (%)**	24.39	25.93	29.30
**ST (%)**	30.14	24.99	23.68

##### 3.1.4.2 Intra-step number analysis

**FIGURE 10:**
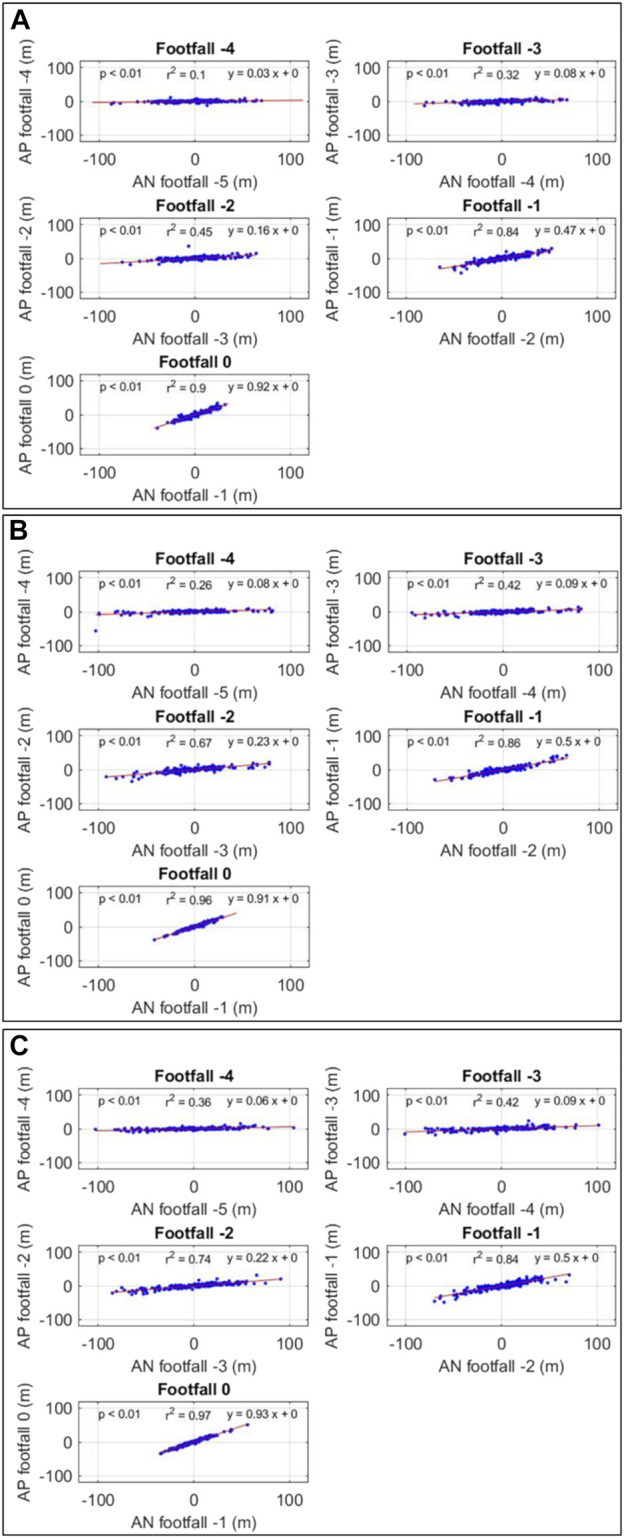
Relationship between the amount of adjustment needed (AN) at a certain footfall and the amount of adjustment produced (AP) in the following footfall. **(A)** real-world condition **(B)** corresponding virtual reality condition **(C)** outdoor virtual reality condition.

Linear regression analyses showed a significant linear relationship between the amount of adjustment needed at a certain footfall and the amount of adjustment produced in the following footfall in the three conditions (Figure 10).

Relationships were significant for all footfalls in the three conditions with increasing slopes (*β*) and *r* ^2^ values. Slopes increase from 0.03 at footfall −4 to 0.91 at footfall 0 in RWC, from 0.08 at footfall −4 to 0.92 at footfall 0 in corresponding VR and from 0.06 at footfall −4 to 0.93 at footfall 0 in outdoor VR. At footfall 0, *r* ^2^ is close to 1 in the three conditions (0.90 in RWC, 0.96 in corresponding VR, and 0.97 in outdoor VR).

In terms of gait parameters, our first hypothesis (H1) was confirmed. We hypothesized that the participants’ gait adaptability behavior is not affected by the type of environment (real-world vs*.* virtual). Although analyses revealed differences of absolute and constant pointing errors between RWC and VRC, these errors reflect a high level of accuracy whatever the type of environment. Analyses showed that the participants adopted the same gait adaptability behavior reflecting the use of a generic control mechanism (based on information-movement coupling) whatever the type of environment.

For the sub-challenge, (H6) was confirmed. We hypothesized that the virtual visual context does not affect gait adaptability behavior. All outcomes were similar between the two VRC.

### 3.2 Psychological variables

#### 3.2.1 Acceptance of the VR-HMD

**FIGURE 11:**
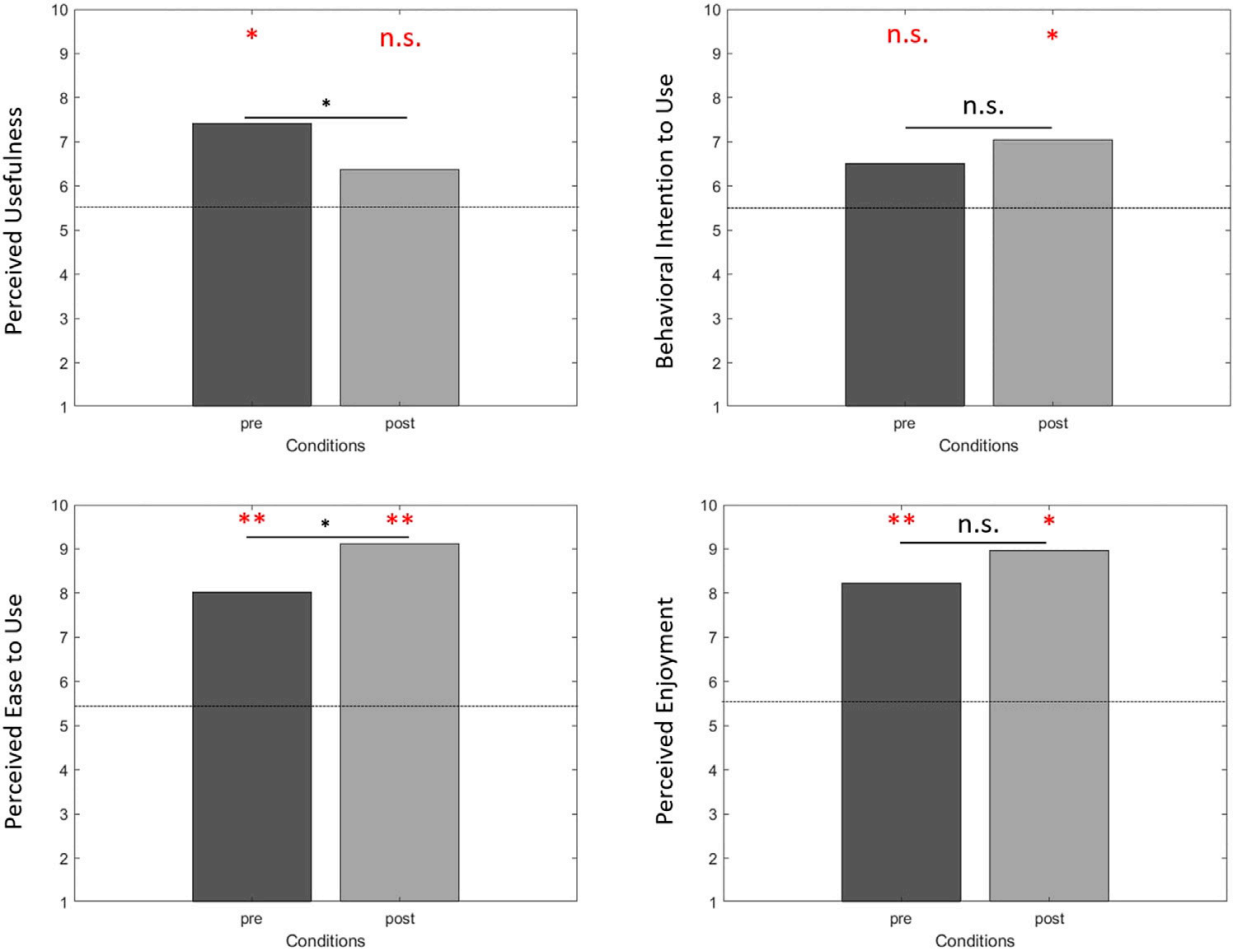
Acceptance of the virtual reality device before (pre) and after use (post). Analyses revealed that only PU after use (*p* = 0.217) and BIU before use (*p* = 0.184) were not different from the mean of the scale (i.e., dotted lines). Analyses showed differences between before and after use for PU (*p* = 0.007) and PEU (*p* = 0.033). n. s. no difference; *p < 0.05; **p < 0.001. In red, compared with the mean of the scale. In black, between conditions analyses.

The results of the different variables used to examine acceptance of the VR-HMD showed that the participants accepted the device before and after use (Figure 11).

Perceived usefulness (PU) was different from the mean of the scale (i.e., 5.5) before use (*p* = 0.006). After use, PU was not different from the mean of the scale (*p* = 0.217). The VR device was considered useful before use but neither useful nor useless after use. Behavioral intention to use (BIU) was not different from the mean of the scale before use (*p* = 0.184). After use, BIU was different from the mean of the scale (*p* = 0.048). The participants did not intend to use this VR device before use but after use they intended to use it. Perceived ease of use (PEU) was different from the mean of the scale before (*p* < 0.001) and after (*p* <0 .001) use. The VR device was considered easy to use before and after use. Perceived enjoyment (PE) was different from the mean of the scale before (*p* <0 .001) and after (*p* = 0.006) use. The VR device was considered enjoyable before and after use.

Perceived usefulness (PU) was high before use (PU_before_ = 7.42) and decreased after use (PU_after_ = 6.37). A Wilcoxon test showed a significant difference (*p* = 0.007). Behavioral intention to use (BIU) was medium before use (BIU_before_ = 6.52) and after use (BIU_after_ = 7.05). A Wilcoxon test showed no difference (*p* = 0.292). Perceived ease of use (PEU) was high before use (PEU_before_ = 8.02) and increased after use (PEU_after_ = 9.12). A Wilcoxon test showed a significant difference (*p* = 0.033). Perceived enjoyment (PE) was high before use (PE_before_ = 8.22) and after use (PE_after_ = 8.97). A Wilcoxon test showed no difference (*p* = 0.183).

#### 3.2.2 Cybersickness and sense of presence

Cybersickness was low in corresponding VR (mean = 2.40, SD = 1.69) and outdoor VR (mean = 2.23, SD = 1.36). Cybersickness was different from the mean of the scale (i.e., 5.5) in corresponding VR (*p* <0 .001) and in outdoor VR (*p* <0 .001). Participants did not experience cybersickness in the two VRC.

Sense of presence was high in corresponding VR (mean = 8.33, SD = 1.97) and outdoor VR (mean = 8.35, SD = 2.24). Sense of presence was different from the mean of the scale (i.e., 5.5) in corresponding VR (*p* < 0.001) and in outdoor VR (*p* <0 .001). Participants felt present in the two VRC.

In terms of psychological variables, our hypothesis (H2) was partially confirmed. We hypothesized that the HMD would be accepted by the participants before its use. For the four different variables used to examine acceptance of the VR device, only BIU was not different from the mean of the scale before use. Our hypothesis (H3) was partially confirmed. We hypothesized an increase in HMD acceptance after use compared with its level before use) but only the PEU increased. Our hypotheses (H4) and (H5) were confirmed. We hypothesized a low level of cybersickness and a high level of sense of presence in both VRC.

## 4 Discussion

In the present study, we sought to ensure that gait adaptability behavior in the elderly was comparable when performing a goal-directed displacement in the real-world and in fully immersive VR. We also ensured that the participants accepted the HMD before and after use. Moreover, we investigated the influence of the visual context on gait adaptability behavior and on acceptance of this VR-HMD. Finally, we studied cybersickness and sense of presence in the two virtual environments.

### 4.1 Gait parameters

#### 4.1.1 Gait speed analysis

In our study, the participants walked on average at 1.07 m/s, 0.96 m/s, and 0.98 m/s (SD = 0.02 m/s) in RWC, corresponding VR and outdoor VR, respectively. They demonstrated a similar gait speed compared with previous studies. Mirelman et al. (2016) found that their participants walked on average at 0.99 m/s during a real-world gait training on treadmill and at 1.00 m/s in low-immersive VR gait training on treadmill. Caetano et al. (2018) found that, during a gait adaptability test in the real world, the participants walked on average at 1.10 m/s (SD = 0.1 m/s) and 0.92 m/s (SD = 0.2 m/s) for low risk and high risk of falling groups, respectively. A low gait speed is a strong predictor for adverse health outcomes such as falls, functional decline, and mortality in older people (Abellan Van Kan et al., 2009) and it is adopted by high-risk older adults to approach the obstacle/target due to cognitive and motor deficits (Caetano et al., 2018). A gait speed below 1.0 m/s indicates an increased fall risk (Kyrdalen et al., 2019). In our study, gait speeds were close to 1.0 m/s in the three experimental conditions. This result can be explained by the experimental set-up. The participants walked with a safety harness connected to an in-ceiling rail by a lanyard and had to overcome inertia during the first steps.

In terms of gait speed comparison between real and virtual environments, we demonstrated no difference between the two types of environments, in line with Janeh et al.’s (2018) study conducted with older adults. Moreover, we found no difference between the two different virtual environments. In summary, in our study, the participants adopted the same gait speed whatever the type of environment and visual context.

#### 4.1.2 Pointing accuracy analysis

We found that the participants had a lower APE in VRC (2.78 cm ± 0.86 cm and 3.20 cm ± 1.66 cm in corresponding VR and outdoor VR, respectively) compared with RWC (4.53 cm ± 1.43 cm). In our study, the participants were very accurate and even more so in VRC. In the three conditions, APEs were smaller than those recorded in the studies in young adults by De Rugy et al. (2000), Montagne et al. (2000), and Cornus et al. (2009) which were 15 cm, 12 cm, and 9 cm respectively. These differences could have also arisen from differences in both task and methodological constraints. In the study by De Rugy et al. (2000), participants walked on a treadmill while the virtual environment was depicted on a screen in front of them, and the target disappeared just before locomotor pointing. The disappearance of the target could have led to performance deterioration. In their long-jumping study, Montagne et al. (2000) asked their participants to jump as far as possible. It is worth mentioning that although good pointing accuracy is a necessary condition for a good jump, the very high running velocity makes accurate pointing difficult (speed-accuracy trade-off). In the study by Cornus et al. (2009), participants had to step over an obstacle, so very precise pointing was not crucial.

Furthermore, we found that the participants had a lower CPE in VRC (−2.47 cm ± 0.98 cm and −2.51 cm ± 2.11 cm in corresponding VR and outdoor VR, respectively) compared with RWC (−3.52 cm ± 1.84). The results showed that the participants tended to place their feet before the target in the three conditions. This result must be explained by the instructions given (i.e., place the right foot tip as close as possible to the lower extremity of the target).

In addition, there was no difference in the APE and the CPE between the VRC. The virtual visual context does not modify the pointing accuracy.

In summary, in our study, the participants had a high level of pointing accuracy in both real and virtual environments.

#### 4.1.3 Inter-trial analyses

The standard deviations of both the step lengths and the toe-obstacle distances during the target approach phase were computed.

First, high values of standard deviations of toe-obstacle distances at the beginning of the target approach phase (i.e., footfall −4 and −3) reflect the fact that the target position was manipulated across trials. Moreover, our results revealed an increase in the mean standard deviation of step lengths at footfall −2 (i.e., three steps before pointing) combined with a decrease in the mean standard deviation of toe-obstacle distances, in the three experimental conditions. These inverted patterns of variability are in agreement with the results obtained in other studies based on different kinds of goal-directed locomotion tasks (Montagne et al., 2000; Cornus et al., 2009; Van Andel et al., 2018a). A precise comparison of our results with the results obtained in these studies revealed that both Van Andel et al. (2018a) and Montagne et al. (2002) also reported an increase in step length variability three steps before pointing, while the increase in variability occurred one step sooner in the study by Cornus et al. (2009). Furthermore, Montagne et al. (2000) and Cornus et al. (2009) reported a decrease in the mean standard deviation of toe-obstacle distances one step sooner than in our study. Rather than emphasizing these slight differences, which probably came from specific task constraints and differences in the methods used, we prefer to focus on the strong similarities in the patterns of variability among the different studies.

These concomitant and inverted patterns of variability could mirror some kinds of functional locomotion adaptations which have been labeled “compensatory variability” by Bootsma & van Wieringen (1990). The increase in step length variability is due to step length adjustments produced to perform an optimal foot positioning (i.e., minimize toe-obstacle distance at the last footfall). Importantly, this “compensatory variability” was not affected by the type of environment nor by the visual context. We found similar patterns of variability whatever the experimental conditions.

#### 4.1.4 Trial-by-trial analyses

##### 4.1.4.1 Inter-step number analysis

In our study, 88.3%, 87.9%, and 88.9% of trials were regulated in RWC, corresponding VR, and outdoor VR, respectively. The percentage of regulated trials (88.6%) was very close to the results of Van Andel et al. (2018a) (87%), but slightly greater than those obtained by Montagne et al. (2000) and Cornus et al. (2009) (77.7 and 65.9% respectively). This result demonstrates that the manipulation of the target position between trials almost always compelled the participants to regulate their displacement.

The participants adopted three different step adjustment strategies, either lengthening or shortening or mixed strategies, similar to those observed by Cornus et al. (2009) and Van Andel et al. (2018a) in pointing tasks and those observed by Montagne et al. (2000) in a long-jumping task. The percentage of mixed trials in our study (47.2% on average) was greater than those reported by Montagne et al. (2000), Cornus et al. (2009), and Van Andel et al. (2018a) (20%, 7.5 and 22% respectively). Among the non-mixed trials, in our study, the participants adopted in the same proportion lengthening trials (50.3% on average) and shortening trials (49.7% on average). However, in Cornus et al. (2009) participants adopted a higher percentage of lengthening strategy than of shortening strategy (61,1 and 38.9% respectively), whereas in Van Andel et al. (2018a) the opposite was shown (37.7% of lengthening strategy and 62.3% of shortening strategy). In our study, the participants did not adopt a specific strategy (either lengthening or shortening) to regulate their displacement, which suggested a trade-off between cautious walks with shortened steps and efficient walks with lengthening steps (Huang et al., 2010). These results indicated that the strategies adopted depend on the characteristics of the investigated population, as shown in the study by Van Andel et al. (2018b).

In summary, the participants adopted similar step adjustment strategies whatever the experimental condition. Strategies of adjustments of step lengths were not affected by the type of environment nor by the visual context.

##### 4.1.4.2 Intra-step number analysis

The intra-step number analysis was conducted to determine the amount of adjustments needed at a given footfall (in relation to toe-obstacle distances) and the amount of adjustments produced during the next footfall (in relation to step lengths) for each footfall.

In our study, the relationship between these two variables was statistically significant from footfall −4 in the three experimental conditions. The slope of the regression lines (*β*) increased over the target approach from 0.06, on average, at footfall −4, to 0.95, on average, at footfall 0. Moreover, the value of *R* ^2^ increased slightly over the target approach in the three experimental conditions from 0.24, on average, at footfall −4, to 0.94, on average, at footfall 0. As in previous studies, the linearity of the relationship increased progressively from footfall −4 to footfall 0. This increasing inclination of the regression lines (i.e., increasing slopes) suggests that during the approach the amount of adjustments produced at a given footfall moves closer and closer to the amount needed (i.e., the amount to be adjusted) to succeed in the task. The current difference of toe-obstacle distance (i.e., the amount of adjustments needed) at a given footfall was rectified by a step length modulation (i.e., the amount of adjustments produced) at the following footfall. Moreover, at the last footfall (i.e., the pointing one), the inclination of the regression line in our study was almost equal to 1 (*β* = 0.94), slightly above that in Cornus et al. (2009) (*β* = 0.84). This result underlines a strong relationship between the two variables, particularly during the last footfall. During this last footfall, the difference of toe-obstacle distance at footfall -1 was almost totally rectified by the step length modulation at the last footfall (i.e., footfall 0). These results demonstrated that the underlying control mechanism is based on a close information-movement coupling. Step length adjustments were produced gradually when needed. Adjustments produced by the participant, within a trial, depend on the current state of the agent-environment system.

In addition, our results revealed that the participants adopted this same gait adjustment behavior whatever the experimental condition. This type of analysis demonstrates the use of a generic control mechanism based on a tight coupling between information and movement and leading to successive adjustments depending on the state of the agent-environment system, in both real and virtual environments. Moreover, we found no differences in control mechanism between the two different virtual environments.

In summary, in our study, we found the control mechanism was not affected by the type of environment nor by the visual context.

### 4.2 Psychological variables

#### 4.2.1 Acceptance of the VR HMD

We aimed to ensure that the participants accepted the VR device before and after its use. First of all, we found that the participants had a high perceived usefulness, perceived ease of use, and perceived enjoyment before use. The participants considered the HMD useful, easy to use, and enjoyable before use, in line with the results of Mascret et al.’s (2020) study. However, the participants did not intend to use the HMD before a first use, but they did not refuse it either. This was not so surprising because neutral attitudes toward HMD prior to a first exposure have already been found in older adults (Huygelier et al., 2019). We also showed that acceptance of the HMD was modulated after use. The participants considered the HMD easier to use and as enjoyable as before use, and they intended to use it after a first use. However, they considered the HMD neither useful nor useless after use. The decrease in perceived usefulness after use may be due to the purpose and the protocol of the present study. At the beginning of the experiment, the presentation of the VR set-up to participants with the short video focused on the role of the HMD in fall prevention, which may explain why perceived usefulness scores were high before a first use of the HMD. But after use, the participants may have considered that the pointing task performed in the study was not effectively useful for preventing fall occurrence, which was not in fact its objective. Nevertheless, perceived usefulness was not low after use; it remained around the mean of the scale. Conversely, after its first use, the participants intended to use the HMD, in line with Huygelier et al.’s (2019) study conducted with older adults. After use, increase in perceived ease of use reflects the fact that older adults feel more able to use this VR device, which is often found in the TAM literature when the technological device has a functioning adapted to the target population. Finally, high levels of perceived enjoyment before and after use highlighted the hedonic perception of the participants during VR exposition, which reinforces the numerous results identified in the literature highlighting that VR is mostly considered a hedonic technology before and after use (e.g., Manis & Choi, 2019).

#### 4.2.2 Cybersickness and sense of presence

We found low levels of cybersickness and, conversely, high levels of sense of presence. Participants did not experience cybersickness and felt present in the two VRC. Participants did not experience potential adverse effects of virtual exposition. This result is very important in the development of our training program when we know that cybersickness negatively predicts intention to use an HMD (Sagnier et al., 2020). Moreover, the notion of the subjective experience of being in a virtual environment (by the study of sense of presence) is essential to ensure ecological validity in virtual environments (Slater, 2009; Birenboim et al., 2021). In terms of user experience, these results confirm the possibility of developing virtual gait training programs in various visual contexts for the elderly.

### 4.3 Limitations and directions for future studies

The present study had some limitations that might be addressed in future research.

First, although the representation only of virtual 3D shoes (representing the feet during the locomotion in VR) leads to similar gait adaptability behavior between real and virtual environments, the inclusion of an embodying avatar in our future gait training in fully immersive VR would be necessary. The representation of the user’s body via an avatar in VR will enhance subjective experience of being in a virtual environment and the ecological validity of our VR environments (Slater, 2009; Grabarczyk & Pokropski, 2016).

Secondly, the healthy older adults involved in our study were relatively young, with a mean age of 68.6 years, and had never fallen. In this present study, we had to take methodological precautions on this specific population because our future training programs will concern only pre-fall participants (healthy older adults with no fall history). However, it would be interesting to extend this study to older adults with high risk of falling. Older adults who have experienced a fall are likely not to have the same gait behavior nor the same subjective experience in VR. Fall history (i.e., any fall event experienced by older adults during a specified period of time) influences older adults’ behavior in VR training (Dermody et al., 2020).

Moreover, these methodological precautions should be taken with each kind of technology used, which is rarely the case in the literature. We ensured that gait adaptability behavior was not affected by the use of the VR technology which we will use in our future training programs. We also ensured that older adults accept the VR technology which we will use in our future training programs. Each VR technology (custom or high-performance devices) can have adverse effects on psychological and/or behavioral parameters. We advise those likely to deploy VR training protocols to do the same in the future.

Finally, concerning the study of psychological variables, although the completion of the questionnaires was anonymous, older adults may be subject to social desirability during self-reported questionnaires (Phillips & Clancy, 1972).

## 5 Conclusion

This study should allow us to take methodological precautions before developing our fully immersive VR training program intended to prevent falls in the elderly. This long-term ambition led us to carry out a multidisciplinary study with our target population. We showed that the gait adaptability behavior of older adults was the same whatever the environment (real vs. virtual) during a goal-directed locomotion task. We also demonstrated that older adults accepted the HMD before and after use, with a low level of cybersickness and a high level of sense of presence. We can now conduct our ambitious interventional study intended to propose the more relevant VR training method to develop gait adaptability in older adults.

## Data Availability

The raw data supporting the conclusions of this article will be made available by the authors, without undue reservation.

## Footnotes

1 A pilot study was conducted to ensure the validity of the VR recording system by a simultaneous recording of the footfalls of 18 young participants engaged in a locomotor pointing task. The validity of the HTC Vive Pro^®^ VR system for gait parameters was tested with the GaitRite^®^ walkway system as a golden standard. The GaitRite^®^ walkway system is a validated gait analysis system (Webster et al., 2005) and has excellent reliability for the quantification of gait parameters of young and older people (Menz et al., 2004). The validity was be assessed by comparing feet position data obtained by the walkway system and the VR system. Two thousand, six hundred and forty position values were recorded simultaneously by the two systems (i.e., 528 pointings x 5 steps). A linear regression analysis with position data collected by both measurement systems was computed to investigate the relationship. Two parameters are crucial to assess the validity of VR recording system: the slope of the line and the coefficient of determination (*r* ^2^). The slope of the line indicates the steepness of a line and represents the rate of change in y as x changes. In our case, a slope very close to 1 would indicate that position data recorded by the two systems are of the same magnitude. R^2^ is a measure of goodness of fit and it is interpreted as the proportion of the variance in the y that is predictable from x. In our case, r2 should be very close to 1 to ensure a strong relationship between the two position data. In our study, the linear regression analysis showed a significant relationship between the position data (*p* < 0.05) recorded by the two systems with a slope of the line of 0.99 and an *r* ^2^ of 1.
