# Supplementary material for: Validation of an immersive virtual reality device accepted by seniors that preserves the adaptive behavior produced in the real world
Source: Front Bioeng Biotechnol. 2022 Sep 2;10:917486. doi: 10.3389/fbioe.2022.917486 (PMC9479106; doi:10.3389/fbioe.2022.917486)
Supplement: Supplementary file 1 [file DataSheet1.docx]

**Supplementary material**

Supplementary Table S1. Slopes (β), R² values and p-values of linear relationship between the amount of adjustment needed at a certain footfall and the amount of adjustment produced in the following footfall for older adults in the three conditions (RW: real-world; VR: virtual reality).

|  | | **RW** | **Corresponding VR** | **Outdoor VR** |
| --- | --- | --- | --- | --- |
| **Footfall -4** | **β** | 0.03 | 0.08 | 0.06 |
|  | **R²** | 0.10 | 0.26 | 0.36 |
|  | **p-value** | <.01* | <.01* | <.01* |
| **Footfall -3** | **β** | 0.08 | 0.09 | 0.10 |
|  | **R²** | 0.32 | 0.42 | 0.42 |
|  | **p-value** | <.01* | <.01* | <.01* |
| **Footfall -2** | **β** | 0.16 | 0.23 | 0.22 |
|  | **R²** | 0.45 | 0.67 | 0.74 |
|  | **p-value** | <.01* | <.01* | <.01* |
| **Footfall -1** | **β** | 0.47 | 0.50 | 0.50 |
|  | **R²** | 0.84 | 0.86 | 0.84 |
|  | **p-value** | <.01* | <.01* | <.01* |
| **Footfall 0** | **β** | 0.92 | 0.91 | 0.93 |
|  | **R²** | 0.90 | 0.96 | 0.97 |
|  | **p-value** | <.01* | <.01* | <.01* |

Supplementary Table S2. Mean, standard deviation (SD) of the four variables of acceptance at the beginning of the experiment (T1) and after the second virtual reality condition (T2). Perceived usefulness (PU), perceived ease of use (PEOU), perceived enjoyment (PE), and behavioral intention to use (BIU).

|  | **T1** | | | **T2** | | |
| --- | --- | --- | --- | --- | --- | --- |
|  | **Mean (SD)** | **One-sample t test (Wilcoxon signed-rank test)** | | **Mean (SD)** | **One-sample t test (Wilcoxon signed-rank test)** | |
|  |  | **V** | **p** |  | **V** | **p** |
| **PU** | 7.42 (2.53) | 178.50 | .006* | 6.37 (2.97) | 138.50 | .217 |
| **BIU** | 6.52 (3.00) | 141.00 | .184 | 7.05 (3.30) | 149.50 | .098 |
| **PEU** | 8.02 (2.12) | 175.00 | .001* | 9.19 (0.71) | 190.00 | <.001* |
| **PE** | 8.22 (2.55) | 191.50 | .001* | 8.97 (1.61) | 207.50 | <.001* |

Note: One-sample t test to compare each variable of acceptance with the mean of the scale.

Supplementary Table S3. Statistics results of the four variables of acceptance between at the beginning of the experiment (T1) and after the second virtual reality condition (T2). Perceived usefulness (PU), perceived ease of use (PEOU), perceived enjoyment (PE), and behavioral intention to use (BIU).

|  | **Wilcoxon signed-rank test** | |
| --- | --- | --- |
|  | **W** | **p** |
| **PU** | 120.50 | .007* |
| **BIU** | 41.00 | .292 |
| **PEU** | 18.00 | .033* |
| **PE** | 8.00 | .183 |

Note: Wilcoxon signed-rank test for paired groups between T1 and T2.

Supplementary Table S4. Mean, standard deviation (SD) of cybersickness and sense of presence in corresponding virtual reality and outdoor virtual reality. Perceived usefulness (PU), perceived ease of use (PEOU), perceived enjoyment (PE), and behavioral intention to use (BIU).

|  | **Corresponding VR** | | | **Outdoor VR** | | |
| --- | --- | --- | --- | --- | --- | --- |
|  | **Mean (SD)** | **Wilcoxon signed-rank test** | | **Mean (SD)** | **Wilcoxon signed-rank test** | |
|  |  | **V** | **p** |  | **V** | **p** |
| **Cybersickness** | 2.40 (1.69) | 4.00 | <.001* | 2.23 (1.36) | 0.00 | <.001* |
| **Sense of presence** | 8.33 (1.97) | 203.00 | <.001* | 8.35 (2.24) | 196.50 | <.001* |

Note: Wilcoxon signed-rank test for paired groups between corresponding VR and outdoor VR.
